# Experimental *Staphylococcus aureus* Mastitis Infection Model by Teat Dipping in Bacterial Culture Suspension in Dairy Cows

**DOI:** 10.3390/ani10050751

**Published:** 2020-04-25

**Authors:** Oudessa Kerro Dego, Paulina A. Pacha, Barbara E. Gillespie, Gina M. Pighetti

**Affiliations:** 1 Department of Animal Science, Knoxville, The University of Tennessee, TN 37996, USA; ppacha@santotomas.cl (P.A.P.); bgillesp@utk.edu (B.E.G.); pighetti@utk.edu (G.M.P.); 2 Facultad de Ciencias Veterinarias, Universidad de Concepción, Chillan 370000, Chile; 3 Escuela de Medicina Veterinaria, Facultad de Ciencias Veterinariasy Recursos Renovables, Universidad Santo Tomás, Temuco 4780000, Chile

**Keywords:** experimental infection, *Staphylococcus aureus*, bovine mastitis, intramammary infection model, teat dipping, bacterial culture suspension

## Abstract

**Simple Summary:**

Udder infection by bacteria such as *Staphylococcus aureus* cause economic losses to dairy production. An effective vaccine is required to control *S. aureus* mastitis. To develop an effective vaccine, a good experimental infection model is required. Infusion of bacteria into the udder can overwhelm the host because it bypasses physical barriers and defense mechanisms in the teat canal. The objective of this study was to develop *Staphylococcus aureus* mastitis challenge model that mimics natural infection. Eight Holstein dairy cows within 1st to 3rd parity at early non-milking period were randomly divided into experimental (*n* = 5) and control (*n* = 3) groups. All teats of experimental cows were challenged by dipping into *S. aureus* culture suspension, whereas those of control cows were dipped into phosphate-buffered saline. Bacteria in the mammary secretion was determined by bacteriological culture. The antibody titer in blood was tested by enzyme-linked immunosorbent assay (ELISA). Other analyses, which include somatic cell count, rectal body temperature, inflammatory changes in mammary secretion, and gland tissues, were assessed. Results showed that three and one of five experimental cows developed subclinical and clinical mastitis, respectively. The remaining cow was infected with *Staphylococcus chromogenes.* In conclusion, experimental *S. aureus* mastitis can be induced by teat dipping in the bacterial culture.

**Abstract:**

Mastitis is inflammation of mammary glands usually caused by bacteria such as *Staphylococcus aureus*. Dairy cows are susceptible to mastitis during early dry and transition periods. Effective vaccine is needed during these periods. One of the limitations to develop an effective vaccine against *S. aureus* is the absence of good infection model. Intramammary infusion (IMIF) with *S. aureus* has been used as an infection model to test vaccine efficacy. IMIF is reliable in causing mastitis, but it bypasses physical barriers, non-specific natural defenses, and immunity in the teat canal. IMIF also transfers a large number of bacteria into the intramammary area at once. The objective of this study was to develop *S. aureus* IMIF model that mimics natural infection. Eight Holstein dairy cows were randomly divided into two groups of experimental (*n* = 5) and control (*n* = 3) cows. All teats of experimental cows were dipped in *S. aureus* culture suspension, whereas that of control cows were dipped in phosphate-buffered saline. Results showed that four of five cows were infected with challenge strain by day 3 of the challenge. The remaining cow was infected with *Staphylococcus chromogenes.* In conclusion, an experimental *S. aureus* intramammary infection can be induced by teat dipping into bacterial suspension.

## 1. Introduction

Staphylococcal mastitis is a major cause of economic losses in dairy production worldwide [1,2]. Coagulase positive *Staphylococcus aureus* is one of the most common contagious mastitis pathogens in dairy cows, with an estimated incidence rate of 43–74% [3,4]. More recently, coagulase-negative *Staphylococcus species* (CNS) such as *S. chromogenes, S. simulans, S. xylosus, S. haemolyticus, S. hyicus* and *S. epidermidis* are increasingly isolated from bovine milk [5,6,7,8] with *S. chromogenes* being the most increasingly diagnosed causative agent of subclinical mastitis. *Staphylococcus chromogenes* [9] and other CNS [10], have been shown to cause subclinical infections in dairy cows that reduced the prevalence of contagious mastitis pathogens. 

Dairy cows are susceptible to mastitis during early dry period and transition (3 weeks before parturition and 3 weeks after parturition) or periparturient periods [11,12] with *S. aureus* being reported as a major pathogen [13]. Current mastitis control measures are based on milking hygiene; use of properly functioning milking machines; maintaining clean, dry, comfortable housing areas; good nutritional programs; segregation and culling of persistently infected animals; dry cow antibiotic therapy; and proper identification and treatment of cows with clinical and subclinical mastitis. When fully adopted and applied; these measures are known to reduce incidence rates of contagious mastitis pathogens including *S. aureus* mastitis; however, because of limited adoption and application of these control measures *S. aureus* mastitis continues to be the most common disease that causes major economic losses in dairy cattle production. 

Therefore, a sustainable intervention tool such as an effective vaccine is required to control staphylococcal mastitis during these critical periods to improve productivity and wellbeing of dairy cows. One of the major constraints affecting the development of an effective vaccine against *S. aureus* mastitis is the absence of uniform and good experimental challenge model that mimics natural intramammary infection (IMI). Intramammary infusion of *S. aureus* is a reliable method in terms of inducing experimental *S. aureus* mastitis [14,15,16]; however, it is an unrealistic infection model since it bypasses physical barriers at teat opening, non-specific natural defenses and inducible innate and acquired immune effectors in the teat canal. Moreover, intramammary infusion overwhelms the host immunological defenses because the number of bacteria infused into the intramammary area during experimental challenge [17,18] is much higher than natural infection. Therefore, a challenge model that is closely similar to natural infection is required for evaluation of the efficacy of an experimental vaccine against *S. aureus* mastitis. The objective of this study was to develop an experimental *S. aureus* mastitis challenge model by teat dipping in a *S. aureus* culture suspension. 

## 2. Materials and Methods 

### 2.1. Study Animals 

This study was approved by the University of Tennessee’s Animal Care and Use Committee (IACUC#=2394-1115). Prior to the enrolment in the study, all cows were tested for the presence of IMI by culturing milk samples and analysis of somatic cell count (SCC). A total of 8 pregnant Holstein dairy cows within 1st to 3rd lactation in the early dry period were divided into two groups of experimental (*n* = 5) and control (*n* = 3) cows (Table 1). All cows were free of IMI and had average composite milk SCC of ≤250,000/mL of milk except one cow, which had an average SCC of 399,750/mL of milk with no bacterial growth from milk. Experimental and control cows were under the same herd management throughout the study and housed at the East Tennessee Research and Education Center-Little River Animal and Environmental Unit (ETREC-LAEU), Walland, Tennessee.

### 2.2. Bacterial Growth Condition

*Staphylococcus aureus* strain UT2 (SAUT2) used as the challenge strain was originally isolated from a dairy cow with mastitis. An aliquot from a frozen vial of *S. aureus* strain UT2 stored at −80 °C in glycerol/Tryptic soy broth (TSB) was inoculated onto Tryptic soy agar (TSA) plates with 5% sheep blood (Becton Dickinson Microbiology System, Cockeysville, MD, USA) and grown overnight (24 h) at 37 °C. After incubation, 3 colonies were inoculated into 1 L of TSB and grown to mid-log phase (OD_600_: 0.4–0.5) achieved the challenge culture concentration of 1 × 10^5^ CFU/mL approximately after 3.5 h of growth at 37 °C.

### 2.3. Experimental Challenge (Infection)

Experimental challenge (infection) started 10 days after drying off and was conducted once daily for 14 days. Prior to challenge, teats were washed thoroughly with water and a mild dish detergent without antibacterial effects and dried with individual paper towel. Culture suspension was aliquoted into 100 mL individual cups per cow and all available teats were challenged by immersing teats in the culture suspension for 15 s. Challenged teats were allowed to air dry for 10 min prior to releasing cows from the parlor. Immediately before and after the challenge, bacterial suspension was cultured to determinate *S. aureus* number using a viable plate count. To ensure parlor disinfection after challenge, floor was disinfected by bleach at 1:10 dilution. On the Ch+15 each quarter of experimental cows were treated by infusion of 10 mL suspension containing 500 mg ceftiofur hydrochloride for dry cows (Spectramast^®^ DC, Zoetis Inc. Kalamazoo, MI, USA).

#### 2.3.1. Clinical Examination of Challenged Cows

During challenge period, severity of local inflammatory changes on mammary secretion and mammary glands (Table 2 and Appendix B), rectal body temperature (Appendix A), somatic cell count (SCC) and bacterial count were monitored and recorded daily. Animals were observed daily for loss of appetite, restlessness, loss of mobility, and unresponsiveness throughout the study time. The macroscopic inflammatory changes in the mammary secretion was scored as 0 = normal, 1 = flakes, 2 = clots, 3 = stringy/watery/bloody (Table 2). Inflammatory changes in the mammary gland tissue were scored as 0 = normal; the udder is pliable, no detection of heat, pain, redness, and/or swelling, 1 = slight swelling; the udder is less pliable, some firmness detected, heat, pain, redness, and/or swelling not necessarily detected, 2 = moderate swelling; the udder is firm, redness and heat detected, discomfort detected, 3 = severe swelling; the udder is very hard, red and hot, noticeable difference compared to other quarters and the cow exhibit signs of irritation (Table 2). In this study, clinical mastitis was defined as a score of 2 for both milk and udder tissue or a score of 3 either in milk or in udder tissue for three consecutive days. A week prior to drying off during the prescreening, subclinical mastitis was defined as somatic cell count (SCC) of >200,000/mL of composite milk from all quarters or >100,000/mL of milk from the individual quarters with positive isolation of the mastitis-causing bacteria from milk for three consecutive days without clinical signs of mastitis. Somatic cell count (SCC) of dry cow secretion rises markedly and may reach up to 2 × 10^7^ cells/mL of dry secretion after first week of dry period due to the recruitment of cells into the involuting gland and the increase in cell number due to reduction in fluid volume of mammary secretion [19,20]. Because of that, after drying off, somatic cell count was not used as one of the criteria to determine infection but the continuous high count of the challenge strain of bacteria from mammary secretion for 3 consecutive days without clinical signs of mastitis was considered a subclinical infection.

#### 2.3.2. Sample Collection

##### Milk or Mammary Secretion and Blood Sample Collection 

The small volume of milk (2–10 mL) was collected 7 days before drying off (D−7), at drying off (D0), and mammary secretion during challenge period at days 0 to 7 (Ch0–Ch+7), day 10 (Ch+10) and, day 14 (Ch+14) of challenge. Individual quarter milk samples were collected on the day of calving (C) and 3 days after calving (C+3) to assess presence of challenge strain of *S. aureus* (Table 3). Samples were collected aseptically in sterile 15 mL tubes, placed on ice and transported to the laboratory. Blood samples were collected 7 days before drying off (D−7), at drying off (D0), immediately before challenge (Ch0) and at days 3, 7 and 14 of challenge (Ch+3, Ch+7, and Ch+14) to determine immunological responses of cows to *S. aureus* infection (Table 3). Immediately after collection, samples were centrifuged at 2500 rpm for 20 min at 4ºC and serum was separated and stored at −20 °C until evaluated by ELISA assay.

##### Milk or Mammary Secretion Somatic Cell Count (SCC) and Bacterial Count

Somatic cell count and bacterial count of milk or mammary secretion samples were determined at the Dairy Herd Improvement Association Laboratory and Tennessee Quality Milk Laboratory (Knoxville, TN, USA), respectively.

Bacteriological culturing was conducted following the National Mastitis Council guidelines as described by Oliver et al. [21]. Briefly, 100 µL of milk was streaked onto Tryptic soy agar with 5% sheep blood (blood agar plates) (Becton Dickinson Microbiology system, Cockeysville, MD, USA) and incubated at 37 °C for 24–48 h until colony growth was observed. Colony characteristics such as morphology, color, and hemolysis pattern on blood agar plates were recorded. Each colony was Gram-stained and Gram-positive cocci were further tested by catalase test to differentiate staphylococci from streptococci. The catalase-positive *Staphylococcus isolates* were further tested by tube coagulase test using rabbit plasma to differentiate *S. aureus* from coagulase-negative *Staphylococcus spp.* (CNS). Those that were catalase-positive and coagulase-positive were identified as *Staphylococcus aureus*.

#### 2.3.3. Pulsed-Field Gel Electrophoresis (PFGE)

Molecular fingerprinting of challenge strain of *S. aureus* that presents in the mammary secretion during the challenge period was assessed using PFGE as described elsewhere [22,23]. Briefly, each bacterial isolate from mammary secretion during the challenge period that was identified as *Staphylococcus aureus* was stocked at −80 °C in Glycerol/TSB. Each isolate was inoculated on a blood agar plate and incubated overnight. A single pure colony was inoculated to 5 mL of Brain Heart Infusion (BHI) broth (Becton Dickinson Microbiology system, Cockeysville, MD) and incubated at 37 °C for 24 h with shaking at 220 rpm. Bacterial concentration was adjusted to an OD_600_ of 0.9 to 1.1 with phosphate-buffered saline (PBS = pH 7.2) using a spectrophotometer (Bio-Rad Laboratories, Hercules, CA). A 200 µL aliquot of the culture was pelleted and re-suspended in 300 µL of Tris-EDTA buffer (pH 8.0). The suspension was mixed with 1.8% (w/v) InCert agarose (Lonza, Rockland, ME, USA) in Tris-EDTA buffer (ThermoFisher Scientific, Waltham, MA), dispensed into the wells of disposable plug mold (Bio-Rad) and digested with lysostaphin (1 mg/mL in 20 mM sodium acetate, pH 4.5; Sigma Aldrich, St. Louis, Missouri). Plugs were washed four times in 4 mL of Tris-EDTA buffer at 37 °C for 20 min. Following the wash step, agar plugs were cut into a 2 × 2 mm size, equilibrated in 1x *Sma*I restriction buffer for 30 min and digested with *Sma*I (10 U/µL, New England BioLabs Inc., Ipswich, MA, USA) in a total volume of 200 µL (3 µL *Sma*I + 197 µL of 1× buffer) at 25 °C for 3 h. A single plug was loaded on to each tooth of 15 combs with the control *S. aureus* strain NCTC 8325 and incubated at room temperature for 20 min. The comb was placed in the gel-casting platform and 1% SeaKem agarose was added and kept at room temperature for 20 min until solidified. Gel electrophoresis was conducted using the CHEF Mapper (Bio-Rad) at the initial switch of 5 s, with a final switch of 40 s and running time for 21 h at 200 V (6 V/cm) at the temperature of 14 °C using ramp angle of 120°. The gel was stained with ethidium bromide (1.25 μg per mL of water, (Invitrogen, Carlsbad, CA) for 25 min and washed twice for 30 min with fresh distilled water. The images were taken using ChemiDoc^®^ Touch Imaging System (Bio-Rad Laboratories, Hercules, CA, USA), exported to GelCompar II software (Applied Maths Inc., Austin, TX, USA)(Bio-Rad Laboratories, Hercules, CA, USA) and saved as a TIFF file. The TIFF images of PFGE were imported and analyzed using GelCompar II software (Applied Maths Inc., Austin, TX, USA). The intra- and inter-gel PFGE runs were normalized using control *S. aureus* strain NCTC8325. The bands ranging from 10 to 674 kb were used for analysis.

#### 2.3.4. Evaluation of Systemic Humoral Immune Responses Against S. Aureus Infection by ELISA

Serum anti-*Staphylococcus aureus* IgG titer was determined using an indirect enzyme-linked immunosorbent assay (ELISA) as described by [24]. Briefly, 96 well polystyrene plates (Immulon^®^ 2 HB) (ThermoScientific, Rochester, NY, USA) were coated with 1 µg/mL of *S. aureus* surface proteins (SASP) in a sodium bicarbonate (NaHCO_3_) coating buffer [0.015 molar (M) Sodium Carbonate (Na_2_CO_3_) and 0.034 M Sodium Bicarbonate (NaHCO_3_) solution of pH 9.6] and incubated overnight at 4 °C. The coating buffer was removed, and plates were washed 5× using an automated 405 touch screen (TS) microplate washer (Biotek instrument Inc, Winooski, VT, USA) with PBS containing 0.05% tween 20^®^ (v/v) (PBS-T, Bio-Rad Laboratories, Hercules, CA, USA) and blocked with PBS-T containing 1% gelatin (W/V) (PBS-TG) for 2 h. The plates were washed 5× with PBS-T and serum was serially diluted four-fold with PBS-TG starting from 1:100 dilution and incubated for 1 h at room temperature. Plates were washed 5× and 100 µL of 1:10,000 diluted (in PBS-TG) horseradish peroxidase-conjugated polyclonal sheep anti-bovine IgG (heavy + Light Chain) (Bethyl Laboratories, Inc. Montgomery, TX, USA) were added and incubated for 1 h at room temperature. After incubation, plates were washed 5× with PBS-T, and 100 µL of ABTS^TM^ horseradish peroxidase substrate (SeraCare Life Sciences, Milford, MA, USA) were added and incubated for 20 min at room temperature. The absorbance was read at a wavelength of 405 nm using a Synergy H1 Microplate reader (Biotek instrument Inc, Winooski, VT, USA). Data were exported to Excel (Microsoft Corporation. Redmond, WA, USA) and the average +2 standard deviations (avg +2 stdev) of the blank row, which received everything except primary antibody (serum), were used to determine the cutoff point for titer calculation. Serum titers were calculated by the intersection of the least-square regression of A_405_ versus the logarithm of dilution.

### 2.4. Statistical Analysis

To assess the effect of experimental *S. aureus* mastitis challenge (infection) by teat dipping in bacterial culture suspension on development of mastitis, systemic humoral responses a mixed model ANOVA was used (SAS 9.4, SAS Institute Inc., Cary, NC, USA). Continuous measures were assessed using a mixed model ANOVA evaluating the fixed effects of teat dip challenge (treatment) or control at certain time points during the challenge (e.g., Ch0–Ch+7, Ch+10, and Ch+14), the interaction of the treatment and day, cow (challenged, control). A significant effect was declared when *p* ≤ 0.05.

## 3. Results

### 3.1. Clinical Examination of Results during Challenge Study

**Figure 1:**
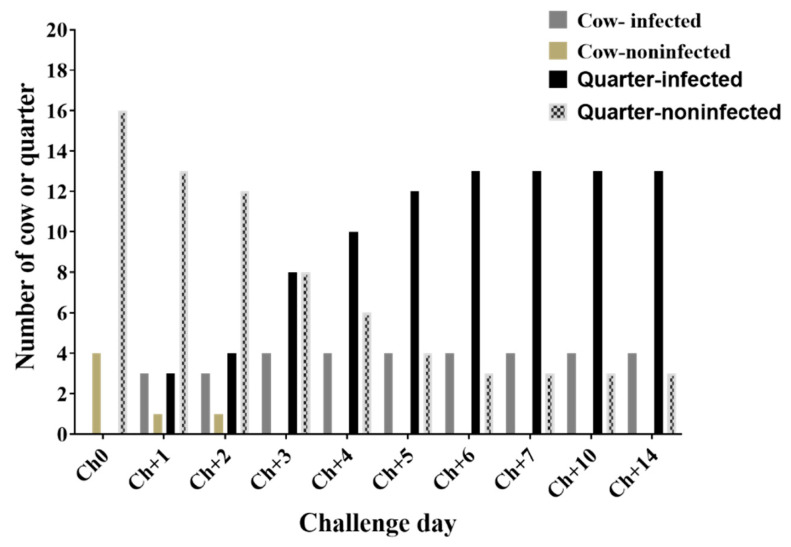
Intramammary infection status of cows or quarters with *S. aureus* during challenge period. Ch0: immediately before the challenge, days 1 to 7 of challenge (Ch+1–Ch+7), days 10 and 14 of challenge (Ch+10 and Ch+14).

**Figure 2:**
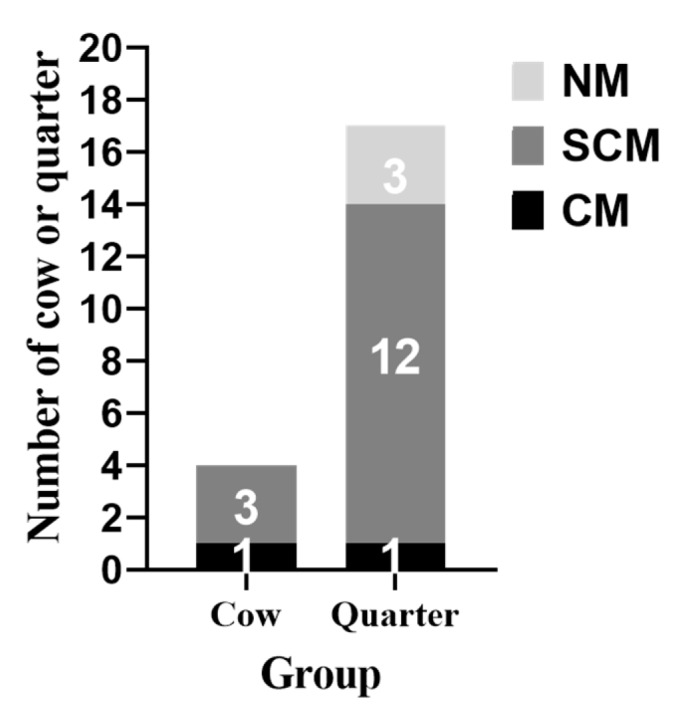
Cow and quarter level infection status. CM: Clinical mastitis, SCM: Subclinical mastitis, NM: No mastitis.

Cow level infection reached 80% (4/5) by day 3 of challenge, whereas quarter level infection reached a maximum of 81.3% (13/16) by day 6 of challenge (Figure 1, Figure 2 and Appendix B). Only one cow out of the four *S. aureus-*infected cows developed clinical mastitis in the left rear (LR) quarter by day 10. The remaining cow from experimental group was infected by *Staphylococcus choromogenes* and exclude from further data analysis. Rectal body temperature was monitored daily throughout the 14 days of the challenge period. No significant differences of mean rectal body temperatures (°C) between the experimental (38.53 °C), and the control (38.59 °C) groups were observed (Appendix A).

### 3.2. Number of S. Aureus Count (CFU/Ml) in the Mammary Secretion

**Figure 3:**
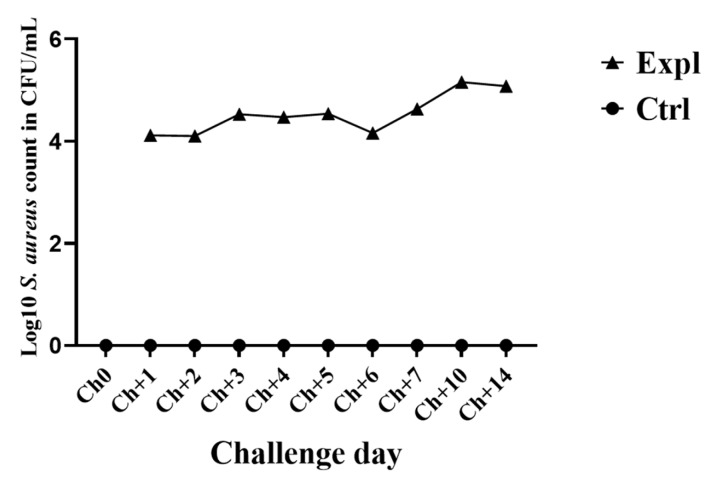
*Staphylococcus aureus* counts (CFU/mL) from mammary secretion during challenge period. ▲: Mean of log10 *S. aureus* count from experimental group, ●: Control group, Ch0: immediately before challenge. Ch+1–Ch+7: days 1 to 7 of challenge. Ch+10 and Ch+14: days 10 and 14 of challenge, respectively.

**Figure 4:**
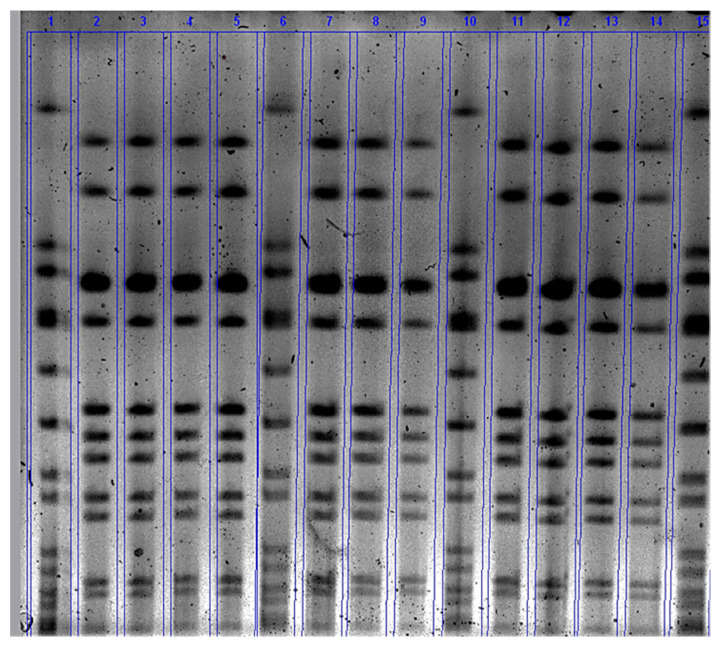
Banding patterns obtained from SmaI digested DNA and pulsed field gel electrophoresis (PFGE) of *S. aureus* strains isolated from mammary secretion from left rear of 4398 cow during challenge period. Lanes 1, 6,10 and 15: *S. aureus* strain NCTC8325 (internal control strain for PFGE analysis); Lanes 2 and 14: *S. aureus* challenge strain UT2; Lines 3, 4, 5, 7, 8, 9, 11, 12 and 13: *S. aureus* isolates identified on day 1, 2, 3, 4, 5, 6, 7, 10 and 14, respectively.

The mean number of *S. aureus* count in mammary secretion ranged from 1.3 × 10^4^ CFU/mL to 1.4 × 10^5^ CFU/ mL at day 1 and 10 of challenge, respectively (Figure 3). All infected quarters shed SAUT2 strain as confirmed by pulsed-field gel electrophoresis results (Figure 4). Bacteriological culture results of milk samples collected at calving (C) and three days after parturition (C+3) was negative for challenge strain of *S. aureus* indicating that post experimental treatment at Ch+15 with antibiotic cleared the infection.

### 3.3. Systemic Immunological Responses of Dairy Cows against S. Aureus Intramammary Infection

**Figure 5:**
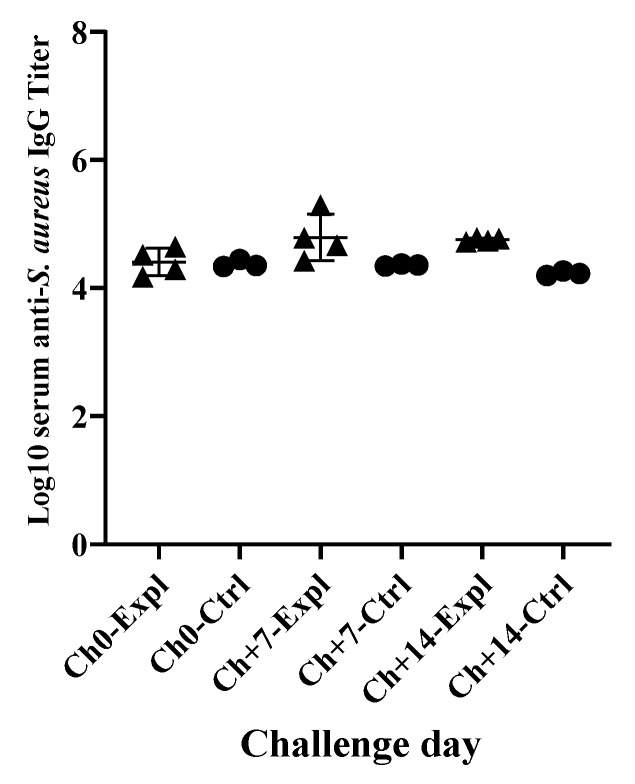
Serum anti-*S. aureus* IgG titers of experimental (Expl: ▲) and control (Ctrl: ●) cows at days 0, 7 and 14 of challenge. No significant difference between experimental and control groups.

There were high background anti-*S. aureus* IgG titers in both control and experimental cows prior to experimental infection. Infection by *S. aureus* induced a slight increase in serum anti-*S. aureus* IgG titers at days 7 and 14 of the challenge but not significantly different from non-infected control cows (Figure 5).

## 4. Discussion

Experimental *S. aureus* intramammary infection was induced by teat dipping into freshly growing bacterial culture suspension at mid-log phase of growth daily. Three cows were infected by day three of challenge in at least one quarter and most quarters by day four of challenge. Despite differences in challenge methods and physiological status of study animals these results are similar to that of Enger et al. [17] who reported that intramammary infusion of *S. aureus* resulted in established intramammary infection (IMI) in 18 of the 19 infused quarters with increased somatic cell count. However, this study challenged pregnant cows at early dry period (14–28 days after drying off) by dipping all available teats in *S. aureus* culture suspension of approximately 1 × 10^7^ CFU/mL of growth media. In their study [17] mammary glands of non-pregnant dry cows after 45 days of drying of were induced to grow by subcutaneous injections of estradiol and progesterone daily for 7 days followed by intramammary infusion of one quarter of each cow with *S. aureus* suspension of 7.5 × 10^3^ CFU/mL. Enger et al. [17] also reported greater number of somatic cell count dominated by neutrophils in *S. aureus* challenged quarters compared with saline infused control quarters.

In this study, at drying off (D0) all cows had somatic cell count (SCC) of less than 250,000 cells/mL of milk except one cow which had average SCC of 399,750 cells/mL of milk with no bacterial growth from milk; however, 14 days after dying off immediately before challenge (Ch0) all cows had high somatic cell count in the range of millions (1 × 10^6^ cells/ mL of dry secretion or greater). There were high counts in the dry secretion of all cows because of recruitment of leukocytes into involuting gland and reduction in the volume of mammary secretion, so somatic cell count was not used to assess infection status. Study by Schukken et al. [16] reported that intramammary infusion of 300–600 CFU/mL of *S. aureus* in the late lactation resulted in 79% (107/135) and 20.7% (28/135) of cows with established and no stablished infection, respectively. Our results indicated that four of five cows (80%) developed established IMI and one cow developed *S. chromogenes* IMI and excluded from data analysis. In another study [25] intramammary infusion of 2.41 to 2.93 log10 CFU of *S. aureus* into two quarters per cow of 68 first lactation Holstein dairy cows resulted in infection of 135 (99%) of 136 challenged quarters. These authors [25] also reported that of 136 quarters challenged, *S. aureus* was not detected in milk from one quarter throughout the entire experimental period of three weeks. In our study, established IMI was developed only in 13 of 16 quarters challenged. Similarly, Bannerman et al. [26] found that intramammary infusion of 10 Holstein dairy cows at mid-lactation with 67 CFU of *S. aureus* into one quarter of each cow resulted in infection of all challenged quarters within 16 h of challenge. These authors [26] reported that somatic cell counts were increased by 24 h post-challenge and remained high throughout the study with eight of ten cows became infected until end of the study on day 8 of challenge. In our study the cow level infection was 80% which is similar to the 80% reported by Bannerman et al. [26]. Using a mildly-virulent strain of *S. aureus,* Eckersall et al. [27] developed experimental subclinical *S. aureus* mastitis by intramammary infusion of 5 × 10^4^ CFU in 10 mL of Ringers buffered salt solution. These authors [27] reported that infusion of two-quarters of each cow at two-time points, at drying off and 28 days after drying off (D0 and D+28) induced subclinical mastitis in eight of 10 infused cows and two cows did not develop mastitis. Petzl et al. [28] induced experimental subclinical *S. aureus* infection in 1st lactation dairy cows at 3 to 5 months post-partum (mid-lactation) by intracisternal inoculation of 10^4^ CFU in 2 mL of 0.9% sterile pyrogen-free saline within 72 h of the challenge.

Most of the challenge infections were induced by intramammary infusion of large number of *S. aureus*. However, in this study, we induced closely similar results by dipping teats in *S. aureus* culture suspension.

The intramammary infusion of *S. aureus* is a well-established method to induce experimental *S. aureus* mastitis in dairy cows [14,15,16,17,25,26,28]. The intramammary infusion of *S. aureus* is a reliable method in terms of causing infection but it is an unrealistic method in terms of mimicking naturally occurring intramammary infections because large number of bacteria are directly delivered into the intramammary area bypassing physical barriers and non-specific natural defenses, as well as inducible innate and acquired immune responses at teat opening and in the teat canal. The intramammary infusion could be a good model to study the pathogenesis of mastitis but not for evaluation of vaccine efficacy against mastitis. Therefore, an experimental challenge model that is closely similar to natural infection is necessary for the evaluation of vaccine efficacy against *S. aureus* mastitis in dairy cows. This teat dipping based infection model is closely similar to natural infection and it is good infection model for evaluation of vaccine efficacy.

## 5. Conclusions

In conclusion, experimental *S. aureus* mastitis can be induced by teat dipping in the bacterial culture suspension without infusing bacteria into the intramammary area. This infection model is good for testing vaccine efficacy because it is closely similar to natural infection.

## Figures and Tables

**Table 1:** Experimental challenge (infection) protocol of *S. aureus.*

Group	No. of Cows	Challenge Strain	Challenge Dose (CFU/mL)	Challenge Route
1	5	SAUT2 *	1 × 10^5^	Teat Dip **
2	3	None	PBS (Control)	Teat Dip **

*: *Staphylococcus aureus* strain 2 from the University Tennessee collection; isolated originally from milk of a cow with intramammary infection. **: Teat dipping into bacterial culture suspension for 15 s.

**Table 2:** Inflammatory changes in dry secretion and mammary gland tissue scoring scheme.

Dry Secretion Appearance	Score	Mammary Gland Appearance
Normal	0	Normal; the udder is pliable. Heat, pain, redness, and/or swelling are not detectable. Cow exhibits no signs of discomfort.
Flakes	1	Slight swelling; the udder is less pliable with some firmness or heavier in weight. Redness, heat, and pain are generally not detectable.
Slugs/clots	2	Moderate swelling; the udder is firm, heavy, reddened and warm to the touch. The cow generally exhibits signs of discomfort (irritable, performs a stepping motion with feet and/or kicks) during evaluation.
Stringy/watery/bloody	3	Severe swelling; the udder is very hard, heavy, red and hot, and noticeably larger than other quarters. The cow is extremely uncomfortable, very irritable and manifests pain by kicking and stepping.

**Table 3:** Sample collection and data recording schedule.

Sample Type	Sample Collection Time (Day)
Milk samples for bacteriological culture and somatic cell count	D−7, D0, C, C+3
Mammary secretion for bacteriological culture and somatic cell count and flow cytometry	Ch0−Ch+7, Ch+10, Ch+14,
Blood samples to measure systemic antibody titers against challenge strain	Ch0, Ch+7 and Ch+14
Rectal body temperature	D−7, D0, Ch0–Ch+14
Recoding scores of inflammatory changes in the mammary secretion and mammary gland tissue	Ch0–Ch+7, Ch+10 and Ch+14

D−: Day before drying off, D+: Day after drying off, D0: Day of drying off, Ch0: immediately before challenge, Ch+: Day of challenge, C: Calving day, C+: Day after calving.

## Appendix A

**Table A1:** Rectal body temperature during the challenge period. G: Group, cow ID: cow identification number (only last two digits were shown for the confidentiality of farm and cow identity), Expl: Experimental Group, Ctrl: Control Group. Ch0: Immediately before challenge. Ch+1–Ch+10: days 1 to 10 of challenge; Ch+14: day 14 of challenge.

Group	Cow ID	Ch+0	Ch+1	Ch+2	Ch+3	Ch+4	Ch+5	Ch+6	Ch+7	Ch+88	Ch+9	Ch+10	Ch+14
**Expl**	−24	38.7	38.6	38.5	38.3	38.5	38.4	38.3	38.4	38.6	38.5	38.2	38.1
	−28	38.4	38.4	38.6	38.4	38.4	38.3	38.6	38.5	38.5	37.7	38.3	38.4
	−98	38.5	38.2	38.1	38.4	38.5	38.3	38.8	38.4	38.8	39	39	39.3
	−94	38.5	38.2	38.6	38.6	38.6	38.6	38.3	38.4	38.7	38.4	38.3	38.6
**Ctrl**	−58	38.6	38.5	38.8	38.7	38.6	38.6	38.5	38.7	38.6	38.7	38.5	38.6
	−71	38.6	38.3	38.6	38.4	38.4	38.5	38.1	38.4	38.3	38.5	38.5	38.7
	−73	38.7	38.7	38.7	38.6	38.6	38.7	38.5	38.7	38.6	38.7	38.4	38.3

## Appendix B

**Table A2:** Score results of inflammatory changes in the mammary glands during challenge period. 0: normal, 1: flakes, 2: clots, 3: stringy/watery/bloody. G: Group, Expl: Experimental Group, CID: Cow Identification number (only last two digits were shown for confidentiality of farm and cow identity), Ctrl: Control Group, Qr: quarter, Ch0: Immediately before challenge, Ch+1–Ch+10: days 1–10 of challenge and day 14 of challenge (Ch+14), RF: right front quarter, RR: right rear quarter, LR: left rear quarter, LF: Left front quarter.

G	CID	Qr.	Ch+0	Ch+1	Ch+2	Ch+3	Ch+4	Ch+5	Ch+6	Ch+7	Ch+8	Ch+9	Ch+10	Ch+14
**Expl**	−24	RF	1	0	0	1	0	0	1	0	1	0	1	1
		RR	0	0	0	1	1	1	1	1	1	1	1	1
		LR	0	1	0	1	1	0	1	1	1	1	1	0
		LF	0	0	0	0	0	0	0	0	0	0	0	0
	−28	RF	0	0	0	0	0	1	0	0	0	0	0	0
		RR	1	1	0	1	1	1	1	1	0	1	1	1
		LR	1	1	0	1	1	1	1	1	1	1	0	1
		LF	1	1	0	0	0	0	0	1	1	0	0	1
	−98	RF	0	0	0	0	1	0	0	0	1	1	1	0
		RR	0	0	0	0	0	1	0	0	1	1	0	0
		LR	0	1	0	0	1	1	1	1	2	2	2	
		LF	0	0	0	0	0	0	0	0	1	0	1	1
	−94	RF	0	0	0	0	0	0	0	0	1	1	0	1
		RR	0	0	0	0	0	0	0	0	0		0	0
		LR	0	0	0	0	0	0	0	0	1	1	0	0
		LF	0	0	0	0	0	0	0	1	0	0	0	0
**Ctrl**	−58	RF	0	0	0	0	0	0	0	0	0	0	0	1
		RR	0	0	0	0	0	0	0	0	0	0	0	0
		LR	0	0	0	0	0	0	0	0	0	0	0	0
		LF	0	0	0	0	1	0	0	0	0	0	1	0
	−71	RF	0	1	0	0	1	0	0	0	0	0	1	0
		RR	0	0	0	0	0	1	1	0	0	0	1	0
		LR	0	0	0	0	0	0	0	0	0	0	0	0
		LF	0	0	0	0	0	0	0	1	0	0	0	0
	−73	RF	0	0	0	1	0	1	0	1	0	0	0	0
		RR	0	0	0	0	0	1	1	1	0	0	1	0
		LR	0	0	0	0	0	0	0	1	0	0	0	0
		LF	0	0	0	0	0	0	0	1	0	0	0	0
